# Gut microbiota shift in Ghanaian individuals along the migration axis: the RODAM-Pros cohort

**DOI:** 10.1080/19490976.2025.2471960

**Published:** 2025-04-06

**Authors:** Barbara J. H. Verhaar, Eva L. van der Linden, Charles F. Hayfron-Benjamin, Ellis Owusu-Dabo, Samuel N. Darko, Sampson Twumasi-Ankrah, Peter Henneman, Erik Beune, Karlijn A.C. Meeks, Max Nieuwdorp, Hilde Herrema, Bert-Jan H. van den Born, Charles Agyemang

**Affiliations:** a Department of Public and Occupational Health, Amsterdam UMC, Amsterdam, The Netherlands; b Department of Vascular Medicine, Amsterdam UMC, Amsterdam, The Netherlands; c Amsterdam Cardiovascular Sciences, Amsterdam UMC, Amsterdam, The Netherlands; d Amsterdam Public Health, Amsterdam UMC, Amsterdam, The Netherlands; e Department of Physiology, University of Ghana Medical School, Accra, Ghana; f School of Public Health, Kwame Nkrumah University of Science and Technology (KNUST), Kumasi, Ghana; g Department of Molecular Medicine, Kwame Nkrumah University of Science and Technology (KNUST), Kumasi, Ghana; h Department of Statistics and Actuarial Science, Kwame Nkrumah University of Science and Technology (KNUST), Kumasi, Ghana; i Department of Human Genetics, Reproduction & Development, Amsterdam UMC, University of Amsterdam, Amsterdam, The Netherlands; j Center for Research on Genomics and Global Health, National Human Genome Research Institute, National Institutes of Health, Bethesda, Maryland, USA; k Department of Experimental Vascular Medicine, Amsterdam UMC, Amsterdam, the Netherlands

**Keywords:** Gut microbiota, migration, urbanization, machine learning

## Abstract

Migration is associated with a substantial change in environmental exposures and health outcomes. We aimed to investigate the shift in gut microbiota composition and the associations with cardiometabolic outcomes in the RODAM-Pros cohort spanning multiple research sites across continents. We determined gut microbiota composition of 1,177 Ghanaian participants in rural Ghana, urban Ghana, and Amsterdam, the Netherlands, using 16S rRNA sequencing. We observed a clear gradient in gut microbiota composition and alpha and beta diversity from rural Ghana to urban Ghana, to Amsterdam. We used pairwise XGBoost machine learning classification models to identify which microbes were most distinct between locations in prevalence and abundance. The associations between these microbes and the locations could partly be explained by differences in confounders such as dietary intake. Groups of microbes that emerged or disappeared along the migration axis were associated with cardiometabolic outcomes, including higher body mass index, higher HbA1c and higher diastolic blood pressure. Concluding, we identified associations between a shift in gut microbiota composition and cardiometabolic risk along the migration axis, underscoring the relevance of gut health in the context of migration-associated adverse health outcomes.

## Introduction

Migration is associated with an increase in cardiometabolic disease, such as an increase in obesity, type 2 diabetes and hypertension prevalence.^1–3^ Explanations for these migration-related adverse health outcomes include lifestyle changes, lower access to healthcare, worse socioeconomic factors and increased psychosocial stress, including discrimination. Another contributing factor is the gut microbiome, which has been established as an important player in cardiometabolic health, by influencing the host immune system, insulin sensitivity, and blood pressure regulation.^4^ Since migration is associated with substantial changes in key factors influencing microbial composition, such as dietary intake and medication use,^5^ migration studies provide valuable insights into gut microbial dynamics. Understanding the associations between gut microbiome shifts associated with cardiometabolic outcomes in the context of migration, could aid in designing targeted interventions to mitigate the migration-associated adverse health outcomes.

Previous research on the effects of urbanization on gut microbiome composition has described a decrease in abundance of microbial families such as *Prevotellaceae* and *Succinovibriaceae*, and an increase in *Bacteroideacae* and *Bifidobacteriacae* with higher urbanization levels.^6–10^ Studies on gut microbiota shifts in the context of migration are less numerous.^11–15^ These studies had smaller sample sizes than urbanization studies, only compared first- and second-generation migrants within the same country, or did not adjust for dietary intake or other relevant confounders. Additionally, the associations of microbial changes with cardiometabolic outcomes remain elusive.

The Research on Obesity and Diabetes among African Migrants prospective (RODAM-Pros) cohort is a well-phenotyped cohort of Ghanaian individuals spanning multiple geographical locations in Ghana and Europe using the same methods of data and sample collection. This presents an opportunity to address the interplay of urbanization and migration with gut microbiome dynamics in shaping health outcomes. We hypothesized that the gut microbiome shift would follow the migration axis, in line with trends in increased cardiometabolic risk along the rural-urban-European gradient. Here, we present evidence of a shift in gut microbiota composition along the migration axis, including the loss and emergence of specific microbes. Our findings contribute to a deeper understanding of the role of microbial dynamics in shaping health disparities between rural and urban settings and upon migration to Europe.

## Materials and methods

### Cohort and participants

We used follow-up data of the prospective population-based RODAM-Pros cohort, collected between 2019 and 2021, including 2165 Ghanaian participants aged over 18 years located in rural and urban Ghana and Amsterdam, the Netherlands. The complete overview of the design and methods of the RODAM-Pros cohort has been published elsewhere.^16^ In brief, the primary aim of the RODAM-Pros cohort is to identify the key changes in environmental exposures and epigenetics that underlie the elevated burden of cardiovascular disease (CVD) risk among sub-Saharan African migrants. In Ghana, at baseline (2012–2015), census data of 2010 were used to draw rural and urban participants in the Ashanti Region, while in Amsterdam the municipality register was used to randomly select and invite Amsterdam-Ghanaians. Those who participated at baseline were invited for follow-up data collection that were used in this analysis. Ethical approval was obtained from the local ethics committees and all participants provided their written informed consent.

For this study, we included 1177 Ghanaian participants of the population-based Research on Obesity and Diabetes among African Migrants Prospective (RODAM-Pros) cohort with available fecal samples and without antibiotic use in the three months prior to sample collection. Data and samples were collected during research visits at rural and urban research sites in Ghana, and urban sites in Amsterdam, the Netherlands, in the period 2019–2021. The research sites in Ghana were located in 15 villages and 11 neighborhoods in two cities (Obuasi and Kumasi), all in the Ashanti region. The majority of Ghanaian migrants located in Amsterdam (98%) were first-generation migrants originating from the same region. For the current analysis, we included 474 subjects in rural Ghana, 460 subjects in urban Ghana and 243 Ghanaian subjects in Amsterdam

Data collection included a general questionnaire, a detailed dietary assessment, physical examination, venous blood draw, and fecal sample collection (described under ‘Gut microbiota composition’). Study visits took place in the morning after an overnight fast. Demographics (including occupation), physical activity, medication use, and medical history were based on self-report. Physical activity was expressed in metabolic equivalent (MET) minutes, for which the intensity level of exercise was multiplied by the duration in minutes. Height and weight were recorded, and BMI was calculated. Blood pressure was measured three times using a semi-automatic oscillometric device, and the average of the last two measurements was used. Hypertension was defined as having a mean systolic blood pressure higher than 140 mmHg or diastolic blood pressure higher than 90 mmHg, or the use of antihypertensive medication. Diabetes was defined as self-reported diabetes, the use of glucose-lowering medication, or a glucose level of ≥7.0 mmol/L during the research visit. Fasting venous blood samples were collected and processed. The aliquoted plasma samples were temporarily stored at −20°C at the local research site, whereafter the samples were shipped to the central laboratories to be stored at −80°C. A ready-to-use colorimetric reagent was used to measure the concentrations of glucose, total cholesterol (TC), high-density lipoprotein (HDL), and triglycerides on an ABX Pentra 400 chemistry analyzer (Horiba ABX SAS, Oberursel, Germany). Low-density lipoprotein (LDL)-C levels were calculated using the Friedewald equation. Glycosylated hemoglobin (HbA1c) was measured by high-performance liquid chromatography (Tosoh G8 hPLC analyzer). Creatinine levels were measured using a kinetic spectrophotometric method (Roche Diagnostics), and estimated glomerular filtration rate (eGFR) was calculated using the CKD-EPI formula.^17^ C-reactive protein (CRP) levels were measured with a particle enhanced immunoturbidimetric assay.

### Dietary data

Dietary intake was assessed using the semi-quantitative Food Propensity Questionnaire (Ghana-FPQ).^18^ The Ghana-FPQ queries for the usual intake frequencies of 134 food items in the 12 months prior to the questionnaire. The Ghana-FPQ is semi-quantitative and does not give information on portion sizes for some of the included food items. Thus, 24 h dietary recalls were conducted in a random sub-sample in each study site (*n* = 251) in order to estimate portion sizes as well as nutrient content of certain recipes. The German Nutrient Database (BLS 3.01) (2010) and the West African Food Composition Table (2012) were used to translate usual food intake into total energy (kcal per day) and macro-nutrient intake (in grams or milligrams).

### Gut microbiota composition

The protocols for fecal sample collections were identical for Ghanaian and Dutch research sites. The study participants were asked to bring morning (within 6 hours) stool samples in a sterile fecal sample tube without preservative (Sarstedt cat.no. 80.734.001) as has been provided by the research team. These samples were transported to the central laboratory and stored at − 80°C. Fecal samples from Ghana were shipped to Amsterdam, the Netherlands, for further processing. DNA was isolated from a 150 mg fecal sample using an adapted repeated bead-beating method,^19^ and purified using the Maxwell RSC Blood DNA kit (Promega). The 16S rRNA gene amplicons were amplified with a single-step PCR protocol targeting the V4 region (515F–806 R primers). After confirmation of PCR products on an agarose gel, the samples were purified using Ampure XP beads (Beckman Coulter), and equimolar pooled. The quality and quantity of the pooled library was checked using High Sensitivity DNA kit (Agilent) on the Bioanalyzer 21,000 (Agilent), and Qubit® dsDNA BR Assay Kit. Next, the samples were sequenced on an Illumina Miseq platform with V4 chemistry and 2 × 251cycles.^20,21^ The reads were processed with a VSEARCH Nextflow workflow (v.0.7.1) that was made publicly available (see code sharing statement). This workflow uses VSEARCH (v.2.27.0) to process the reads and infer amplicon sequence variants (ASVs). Paired-end reads were merged, allowing for a maximum of 30 differences, and then quality filtered with a maximum of 1 expected error. The resulting reads were dereplicated, whereafter ASVs were inferred through denoising with the UNOISE3 algorithm (minimum size 10, alpha 1.5). The ASVs were sorted and singleton ASVs were discarded, as these are most likely spurious. All merged reads were mapped against the ASVs to create a count table. The *assignTaxonomy* (minBoot 50) and *addSpecies* functions from the DADA2 R package (v.1.30.0) were used to assign taxonomy to the ASVs, with the SILVA databases (v.138.1) as reference. MAFFT (v.7.520) was used for sequence alignment, followed by the construction of a phylogenetic tree using FastTree (v.2.1.11) with a generalized time-reversible model (GTR). Phyloseq (v.1.44.0) was used to integrate the ASV table, taxonomy, tree and reference sequences. The count table was rarefied to 15,000 counts per sample. Of 1219 samples, 10 had insufficient counts and were excluded at the rarefying stage, and 32 used antibiotics and were excluded, resulting in a dataset with 1177 samples and 3176 taxa. The taxonomic assignment was available up to the family level for 77.8%, up to the genus level for 50.7%, and up to the species level for 12% of the taxa. At the species level, this proportion of taxa accounted for on average 41% of the microbiota composition.

### Machine learning and statistics

Differences in participant characteristics between the three groups (rural Ghana, urban Ghana and Amsterdam) were tested using analysis of variance (ANOVA) for continuous variables with normal distributions, Kruskal-Wallis tests for continuous variables with non-normal distributions and chi-square tests for categorical variables using the tableone R package (v.0.13.2). To assess differences in dietary intake between geographical sites, we performed a principal component analysis after scaling the five macronutrient groups (*prcomp* function in R).

To evaluate the associations between various host and dietary factors with the gut microbiota composition, we used XGBoost machine learning models to predict these factors from gut microbiota composition. XGBoost is an ensemble method based on extreme gradient boosting, which combines the predictions of decision trees to improve predictive accuracy. Due to its tree-based design, XGBoost can handle the heteroskedasticity, zero-inflation and collinearity that characterize microbiome data relatively well. It is widely recognized for its accuracy and efficiency in predicting outcomes across various omics analyses, including microbiome studies.^22^ Prior to these analyses, we filtered the ASV table for microbes with at least 10 counts in 30% of subjects. The remaining ASVs were used as predictors, while dietary macronutrient groups and other covariates were used as outcome in these models (one outcome per model). We used the XGBoost algorithm in a nested-cross validation design and 200 iterations. The machine learning design is visualized in Supplementary Figure S1. In each iteration, the data was randomly split in a train (80%) and test (20%) set. The hyperparameter grid was subsequently trained on the train set using 5-folds, and the resulting model was tested once on the test set. In each iteration, two random variables were added to serve as benchmarks, and all models were also run with permuted outcome labels to monitor if the model tended to overfit. For regression models (continuous outcomes), we used the resulting explained variance as main model metric, while for classification models (binary outcomes), we used the area under the curve (AUC) as main model metric. In Supplementary Table S1, we provided an overview of all XGBoost models that were used in this study, including the predictors, outcomes and model types used.

To assess differences between rural Ghana, urban Ghana and Amsterdam, we used XGBoost machine learning models with an identical design, to predict location from the microbiota abundance table. These classification models were performed in a pairwise manner (rural versus urban Ghana; urban Ghana versus Amsterdam), since we hypothesized that the gut microbiome shift would follow the migration axis, in line with trends in increased cardiometabolic risk along the rural-urban-European gradient. Next, we used linear regression analyses to assess the effect of site (binary variable) on the highest ranked ASVs (log10-transformed) from the machine learning models to obtain effect sizes. These models were also performed pairwise, comparing rural to urban Ghana, and urban Ghana to Amsterdam. We used three models per ASV; an unadjusted model, a model adjusted for age, sex, BMI and hypertension, and a model that was additionally adjusted for the first two principal components of the dietary principal component analysis. An overview of the regression models that were performed is provided in Supplementary Table S2. The p-values were adjusted using the false-discovery rate and reported as q-values, which were considered significant at < 0.05.

For the prevalence analyses, we transformed the abundance table to a presence-absence matrix, and filtered for ASVs that were prevalent in a minimum of 10% of subjects. We then performed machine learning analyses similar to the abundance analyses, predicting site (rural vs urban; urban vs Amsterdam) from the prevalence of microbes. We selected the 10 highest ranked ASVs from these two models to assess how the prevalence of these microbes differed between the sites. We then assembled two groups based on the absence of the 13 disappearing microbes (vanish versus controls) and on the presence of 6 microbes that emerged (blossum versus controls). The vanish and blossum groups were named after the VANISH and BLoSSUM acronyms used in previous papers on the emergence and disappearance of microbes with westernization.^23,24^ To test the differences between vanish and blossom groups versus controls, we used Mann-Whitney U tests for continuous variables and chi-square tests for categorical variables. P-values were considered significant at < 0.05.

### Data availability statement

Data are available upon reasonable request. These requests can be made to de RODAM-Pros cohort coordinator Dr Eva van der Linden (e.l.vanderlinden@amsterdamumc.nl) or principal investigator, Professor Charles Agyemang (c.o.agyemang@amsterdamumc.nl). The 16S rRNA sequencing data was made publicly available on the European Genome-Phenome Archive (EGA) with identifier EGAD50000001182, https://ega-archive.org/studies/EGAS50000000805.

### Code availability

All code of the bioinformatic pipeline for sequence data processing (https://github.com/barbarahelena/vsearchpipeline.), machine learning and statistical analyses (https://github.com/barbarahelena/rodam) has been shared in public Github repositories. A conda environment yaml for the XGBoost machine learning models, and renv lockfile for the analyses in RStudio were also shared in these repositories. The version of the Nextflow VSEARCH pipeline that was used for this project (v.0.7) can also be found at Zenodo (https://doi.org/10.5281/zenodo.10553479).

## Results

### Population characteristics

We included 1,177 Ghanaian participants, of which 474 subjects resided in rural Ghana, 460 subjects in urban Ghana and 243 Ghanaian subjects in Amsterdam (Table 1). The majority of these subjects were female (64.9%), and the study population was on average 53.5±11.5 years old. Smoking prevalence was very low in all groups, in line with Ghanaian population data.^25^ Cardiovascular risk factors such as BMI, cholesterol, HbA1c, presence of hypertension, and presence of diabetes were lowest in the rural population compared to the other groups, and highest in the Amsterdam Ghanaians. Especially the BMI differences between groups were large, with almost 6 kg/m^2^ difference between rural Ghanaians and Amsterdam Ghanaians. Systolic blood pressure was comparable between the groups, even after exclusion of subjects using blood pressure lowering drugs (22.5%), while diastolic blood pressure was slightly higher in the Amsterdam Ghanaians.

**Table 1.** Population characteristics.

	Rural Ghana	Urban Ghana	Amsterdam	*p-value*
*n*	474	460	243	
Age, years	54.4 ± 12.9	52.6 ± 10.6	53.6 ± 10.0	0.059
Women	292 (61.6)	326 (70.9)	146 (60.1)	0.003
BMI, kg/m^2^	23.0 ± 4.8	28.3 ± 5.7	29.2 ± 4.4	<0.001
Current smoking	6 (1.3)	0 (0.0)	9 (3.8)	<0.001
Systolic BP, mmHg	131.9 ± 24.4	131.9 ± 21.1	133.3 ± 18.4	0.661
Diastolic BP, mmHg	77.4 ± 12.8	78.7 ± 12.6	81.8 ± 10.5	<0.001
Total cholesterol, mmol/L	4.3 ± 1.3	5.1 ± 1.3	5.1 ± 1.0	<0.001
LDL, mmol/L	2.7 ± 1.1	3.5 ± 1.1	3.1 ± 0.9	<0.001
HbA1c, mmol/mol	33.2 ± 10.6	41.9 ± 16.4	40.0 ± 9.7	<0.001
eGFR, mL/min	94.0 ± 16.5	91.9 ± 17.6	84.4 ± 15.3	<0.001
CRP, mg/l	4.3 ± 10.8	3.0 ± 5.3	2.2 ± 2.9	0.002
Hypertension	170 (36.3)	215 (47.0)	152 (62.6)	<0.001
BP-lowering drugs	53 (11.2)	114 (24.8)	98 (40.3)	<0.001
Diabetes	31 (6.6)	54 (11.7)	40 (16.5)	<0.001
Glucose-lowering drugs	13 (2.7)	32 (7.0)	27 (11.1)	<0.001
Lipid-lowering drugs	4 (0.8)	3 (0.7)	32 (13.2)	<0.001
Total calories, kcal/day	2461 ± 963	2256 ± 640	2678 ± 919	<0.001
Carbohydrates, g/day	364 ± 164	306 ± 96.1	321 ± 118	<0.001
Proteins, g/day	68.2 ± 29.0	75.6 ± 22.6	96.8 ± 30.2	<0.001
Fat, g/day	80.0 ± 33.3	80.3 ± 29.8	103 ± 49.8	<0.001
Fibre, g/day	40.1 ± 15.4	36.1 ± 11.2	40.5 ± 14.2	<0.001
Sodium, g/day	2.2 ± 0.9	3.3 ± 1.0	2.9 ± 1.0	<0.001
Alcohol, g/day	0.1 ± 0.5	0.0 ± 0.2	0.5 ± 1.2	<0.001
Physical activity, MET-minutes/week	3500 [900, 8640]	6440 [2160, 10800]	1920 [600, 5520]	<0.001
Occupation, manual work	368 (88.5)	277 (65.8)	78 (72.2)	<0.001

Data is presented as mean±SD, median [interquartile range] or number (percentage). Group differences were tested with analysis of variance (ANOVA) for continuous variables with normal distributions, Kruskal-Wallis tests for continuous variables with nonnormal distributions and chi-square tests for categorical variables. BMI = body mass index, BP = blood pressure, LDL = low-density cholesterol, HbA1c = glycosylated hemoglobin, eGFR = estimated glomerular filtration rate, CRP = C-reactive protein, MET=metabolic equivalent time.

### Shift in gut microbiota composition from rural Ghana, to urban Ghana and Amsterdam

**Figure 1.**
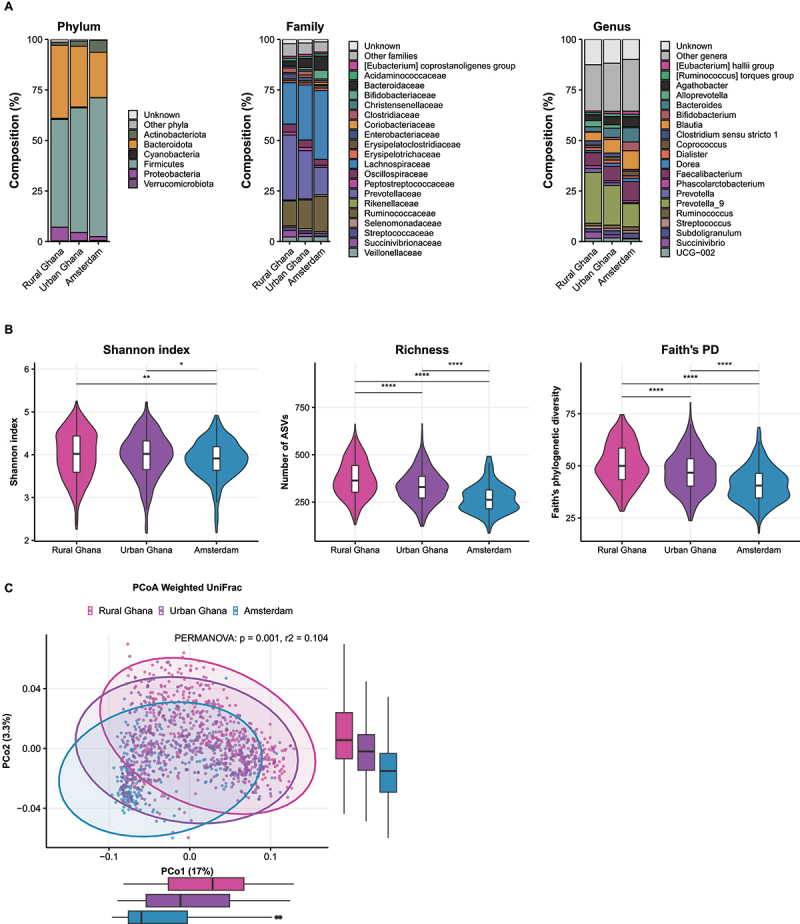
Gut microbiota composition across rural Ghanaians, urban Ghanaians in Ghana and Amsterdam Ghanaians in the Netherlands. a. Relative abundances compared between the three groups at phylum, family and genus level. b. Comparison of Shannon index, richness and Faith’s phylogenetic diversity (PD) between the three groups tested with Mann-Whitney U tests. *p < 0.05, **p  < 0.01, ****p < 0.0001. c. Beta diversity as measured with weighted UniFrac between the three groups.

When looking at the gut microbiota composition descriptively, we could observe a clear shift between rural Ghana, urban Ghana, and Amsterdam (Figure 1(a)). These gradients were visible at phylum, family and genus level. *Prevotellaceae* spp. were most abundant in rural Ghanaians, while *Lachnospiraceae* and *Bacteroidaceae* spp. were most abundant in Amsterdam Ghanaians. There were also group differences in alpha diversity (Figure 1(b)): Shannon index, species richness and Faith’s phylogenetic diversity were all highest in the rural group and lowest in the Amsterdam Ghanaians. In addition, beta diversity as measured with weighted UniFrac distances showed a significant group difference (Figure 1(c); PERMANOVA *p* = 0.001, *r*^*2*^ = 0.10).

### Effect of diet and other covariates on the gut microbiota composition

**Figure 2.**
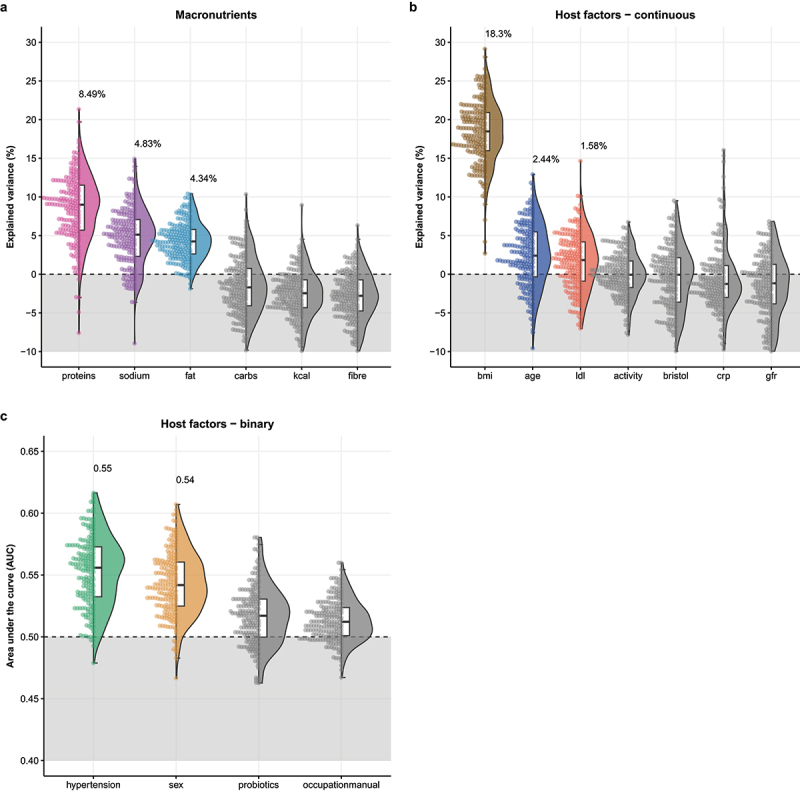
Results from machine learning models with the XGBoost algorithm to predict dietary and host factors from the gut microbiota composition. a. Macronutrient groups predicted using machine learning regression model (shown is the explained variance as main model metric), b. Continuous covariates assessed using the same models, c. Binary covariates assessed using a classification model (shown is the area under the curve (AUC) as main model metric). LDL = low-density cholesterol, CRP = C-reactive protein, GFR = estimated glomerular filtration rate, BPmed = blood pressure-lowering medication.

Next, we aimed to assess which dietary macronutrient groups and other covariates were associated with gut microbiota composition in this cohort. Using a principal component analysis of the macronutrient intake, we found differences in dietary intake between locations, which was for instance characterized by a higher salt and protein intake in urban compared to rural sites (Supplementary Figure S2). Next, we performed XGBoost machine learning models to study the associations between potential confounders and the gut microbiota composition (not stratified for location; Figure 2(a–c)). We did not include smoking or alcohol use in these analyses since the prevalence of both was very low. Of the dietary macronutrient groups, the gut microbiome showed the strongest associations with protein, salt and fat intake (mean explained variance: 8.5%, 4.8% and 4.3% respectively), but no associations with the other components (fibers, carbohydrates, total calories). In addition, we found a strong association of gut microbiota composition with BMI (explained variance 18.3%), and moderate associations with age (explained variance 2.4%) and LDL levels (explained variance 1.5%). We observed lack of associations, or very weak associations, between gut microbiota composition and established determinants of microbiome composition such as sex, fiber intake, physical activity, CRP, and eGFR.

### Identifying the most distinct microbes between rural and urban locations

**Figure 3.**
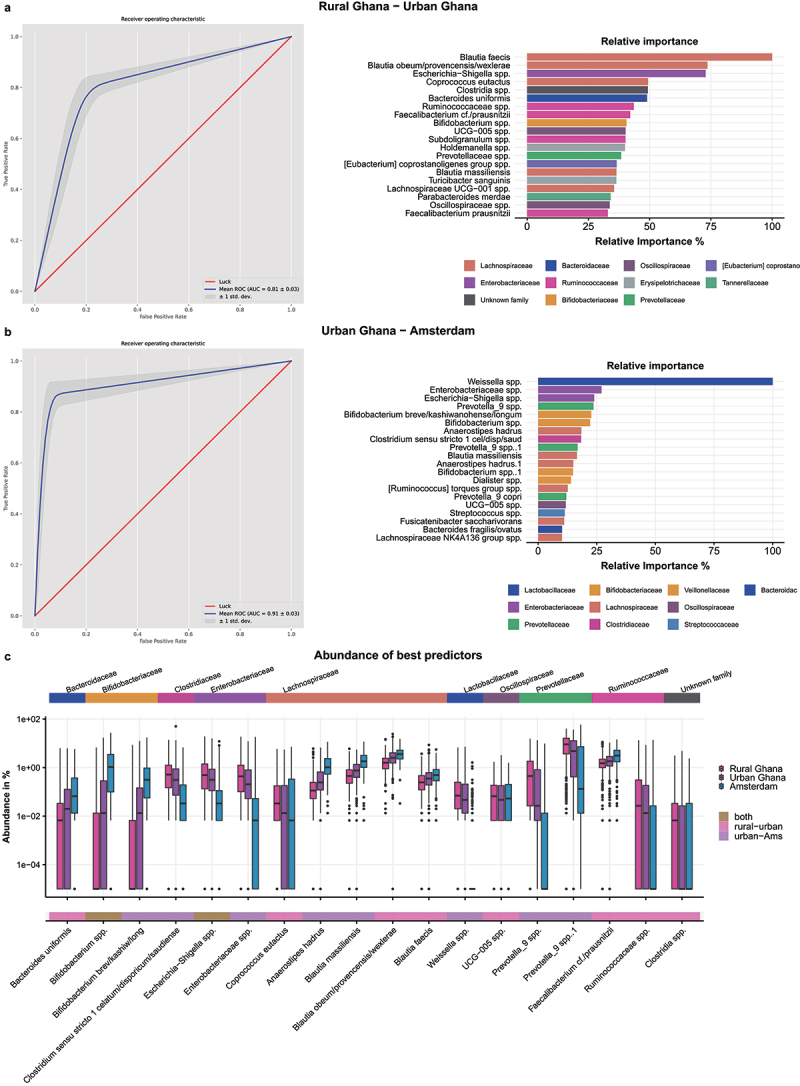
Machine learning prediction models between a. rural Ghanaians and urban Ghanaians residing in Ghana, and b. urban Ghanaians residing in Ghana and Amsterdam Ghanaians in the Netherlands. On the left, the receiver-operating curve resulting from the machine learning model is presented, while on the right, the relative importance of the top 20 features of this model is shown. The colors in the feature importance plots refer to phylogenetic families of assigned taxonomies. c. The relative abundance (%) per location of the 10 highest ranked predictors combined (two ASVs were overlapping, hence in total 18 ASVs), on a log10-scale. The top annotation shows the assigned taxonomic family of the ASVs and the bottom annotation shows from which machine learning model top 20 ranking (rural-urban, urban-Amsterdam or both) the feature originated.

To identify the most distinct microbes between participants from rural Ghana, urban Ghana and Amsterdam, we performed XGBoost machine learning classification models to predict location from gut microbiota composition comparing two locations at a time. The machine learning model to distinguish rural Ghanaians from urban Ghanaians had an area under the curve (AUC) of 0.81, while the model to distinguish urban Ghanaians from Amsterdam Ghanaians had an AUC of 0.91 (Figure 3(a–b)). The models with permuted labels did not perform better than chance (AUC 0.53 and 0.52), indicating that the predictions in the actual models were unlikely to be overfitted. The 20 highest ranked microbes for these two predictions were diverse and included microbes from 17 phylogenetic families (Figure 3(a–b)). *Prevotella* spp. were generally less abundant in urban versus rural Ghana, and lower in Amsterdam compared to urban Ghana, including two *Prevotella_9* spp., with one exception, *Prevotella stercorea*, which was more abundant in Amsterdam compared to urban Ghana (Figure 3(c)). In addition, *Enterobacteriaceae*, including *Escherichia-Shigella* spp., decreased along this gradient. In contrast, *Lachnospiraceae* spp. tended to become more abundant along the rural-urban-Amsterdam gradient. The most profound shift was observed in *Weisella* spp., a microbe of the *Lactobacillaceae* family, which was present in rural and urban Ghana, but virtually absent in Amsterdam Ghanaians.

### Gut microbiota shift is only partly explained by confounders of microbiome composition

**Figure 4.**
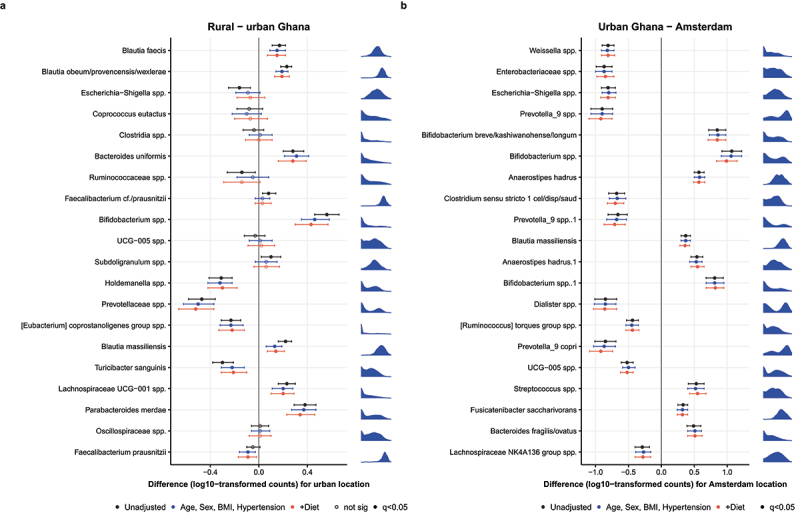
Linear regression models for the most distinct ASVs (microbes) between a. rural and urban Ghana and b. urban Ghana and Amsterdam. Site was used as determined and the log10-transformed counts of the ASVs as outcome. Three models are shown: 1. unadjusted, 2. adjusted for age, sex, BMI and hypertension, and 3. additionally adjusted for diet (the first two principal components). On the right of each plot, the log10-transformed distribution of the ASVs is shown.

Since a range of confounders of microbiome composition could explain the associations between location and the highest ranked microbes from the machine learning models, we used unadjusted and adjusted linear regression models with site (dichotomous) as determinant and the log10-transformed microbial abundances as outcomes. The adjusted models included covariates such as age, sex, BMI, and hypertension, and the first two principal components of the macronutrient intake (Figure 4(a–b)). Of the predictors for rural versus urban location, five associations identified by the machine learning models were not significant in the linear regression models, which could be explained by the nature of the machine learning model, which could also detect non-linear associations. Some of the other rural-urban predictors lost significance after adjustment, such as *Escherichia-Shigella* spp., *Ruminococcaceae* spp., *Subdoligranulum* spp. and *Faecalibacterium prausnitzii*. Most of the other associations were slightly attenuated after addition of the covariates but remained significant. The predictors for urban versus Amsterdam location were all significantly associated with location in the regression model (Figure 4(b)). These associations showed larger differences between urban Ghana and Amsterdam than between rural and urban Ghana, and were much less affected by addition of covariates to the regression models, in line with the higher AUC of the machine learning model.

### Disappearance of microbes along the migration axis is associated with cardiometabolic health

**Figure 5.**
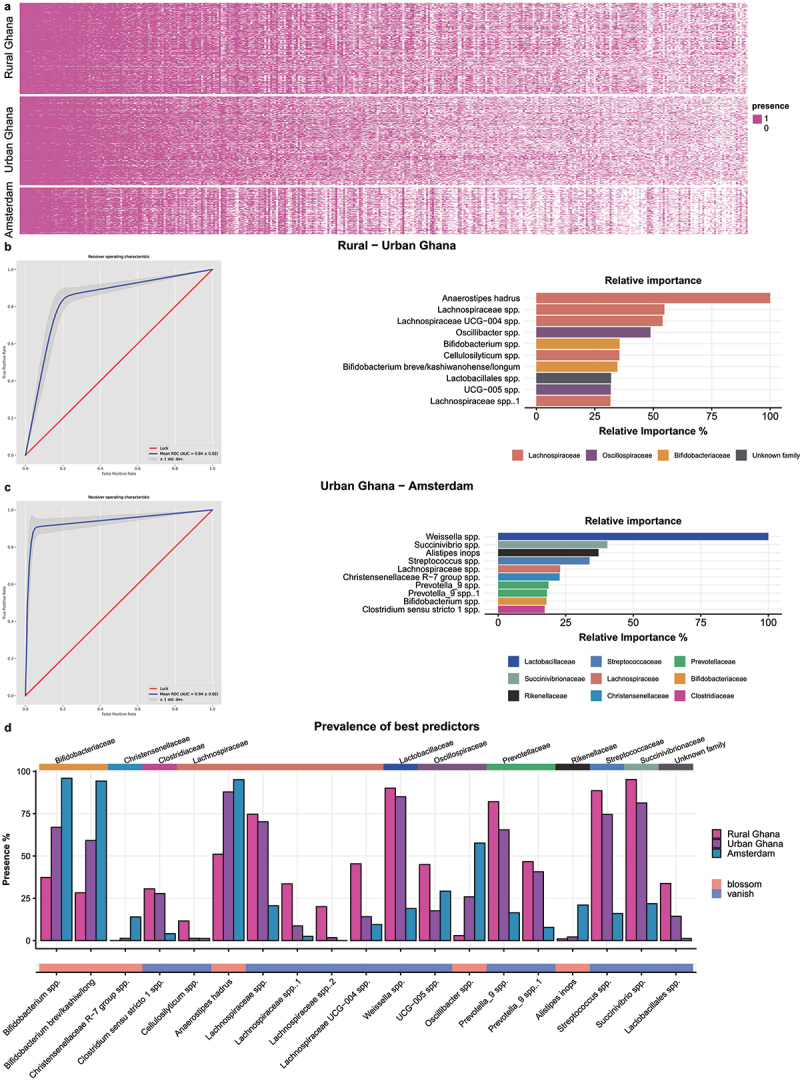
Machine learning results of models to predict site from presence-absence matrix of the microbiota. a. Heatmap of the presence and absence of all ASVs, all colored fields indicate an abundance greater than zero (i.e. presence of the ASV). b. AUC of rural-urban model (left) and highest ranked predictors (right); c. AUC of urban-Amsterdam model (left) and highest ranked predictors (right); d. prevalence of combined best predictors of these model (1 overlapping, hence in total 19 microbes) in different sites. The top annotation shows the phylogenetic family of the ASV while the bottom annotation shows whether the ASV decreases (vanish) or increases (blossom) from rural Ghana to Amsterdam.

**Figure 6.**
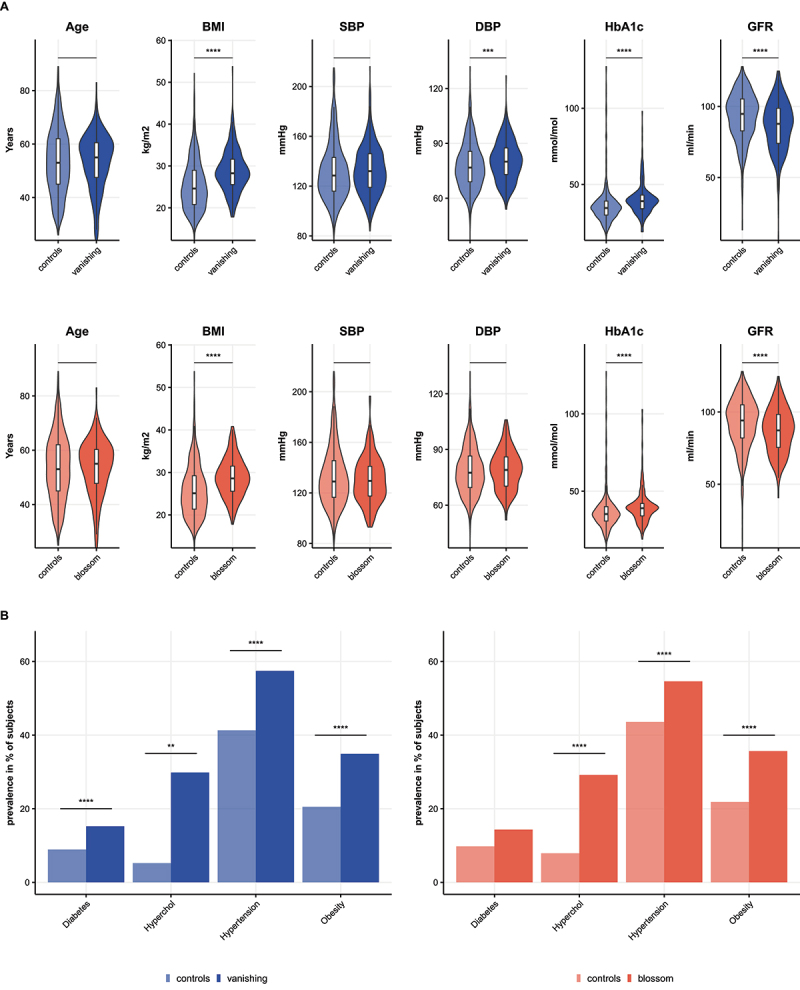
Differences in health outcomes between vanish and control groups and blossom and control groups. a. Differences in continuous variables between controls and vanish (first row) or blossom (second row) groups, tested with Mann-Whitney U tests. b. Differences in dichotomous variables; prevalence of diabetes, hypercholesterolemia, hypertension and obesity is shown for controls versus vanish groups (left) and controls versus blossom groups (right), tested with chi-square tests. BMI = body mass index, SBP = systolic blood pressure, DBP = diastolic blood pressure, GFR = estimated glomerular filtration rate. Significance levels: ** = p < 0.01, *** = p < 0.001, **** = p < 0.0001.

We set out to investigate whether certain microbes disappear from one context to another as a consequence of environmental differences. In previous publications, these groups have been referred to as Volatile and/or Associated Negatively with Industrialized Societies of Humans (VANISH) for disappearing microbes, and Bloom or Selected in Societies of Urbanization/Modernization (BLoSSUM) for emerging microbes.^23,24^ To investigate this in the current study, we transformed the abundance table into a presence-absence matrix (with all values greater than zero labeled as ‘present’), which further emphasized the differences in richness between groups when visualized in a stratified heatmap (Figure 5(a)). Then, we again used XGBoost machine learning models to predict location from the prevalence of microbes. This presence-absence analysis is expected to be more sensitive to changes in low-abundant ASVs. These machine learning models also resulted in very high AUCs (rural-urban AUC 0.84, urban-Amsterdam AUC 0.94). Among highest ranked 19 predictors (10 from the rural-urban prediction and 10 from urban-Amsterdam prediction, with one ASV overlapping; Figure 5(b–c)), 13 microbes (labeled blue in Figure 5(d)) were less prevalent in urban Ghana and/or Amsterdam compared to the rural environment. Next, we divided all participants in two groups based on the absence (at least 10 out of 13) of this collection of microbes, resulting in a group of 315 (“vanish”) versus a group of 862 with these microbes (“controls”). Participants without these microbes appeared to have more cardiovascular risk factors such as higher BMI, higher diastolic BP, and lower eGFR compared to participants with these microbes, despite being of similar age. In addition, the prevalence of hypertension and diabetes was higher in the group without these microbes (Figure 6). Conversely, six of the 20 microbes had a lower prevalence in the rural location compared with Amsterdam (labeled pink in Figure 5(d)). Based on the presence (at least four out of six) of this group of microbes, participants were divided into two groups; 216 (“blossum”) versus 961 (“controls”). Participants with this collection of microbes (“blossum”) had more unfavorable cardiovascular risk profiles (Figure 6). Comparing the dietary intake of the vanish between groups, we found that both the vanish and blossom groups reported a higher intake of protein, fat, and sodium compared to controls (Supplementary Figure S3 and S4). In summary, these data show that groups of microbes that emerge or disappear along the migration axis are associated with differences in dietary intake and cardiometabolic risk factors.

## Discussion

We found a clear shift in gut microbiota composition along a gradient of urbanization and migration among Ghanaian individuals. This shift was evidenced by a lower alpha diversity, group differences in beta diversity and specific changes in microbiome composition. The observed microbial shift had a very diverse nature, both in abundance and prevalence analyses. We investigated the impact of microbiota confounders in this cohort and found that BMI and macronutrient groups such as protein, salt and fat, were strongest associated with gut microbiota composition. However, these covariates did not explain the microbial associations with location (rural-urban-Amsterdam). We additionally showed that the disappearance and emergence of microbes were associated with cardiovascular risk factors, underscoring the relevance of our findings in the relation to migration-related health outcomes.

In the existing literature, different groups of disappearing and emerging microbes have been described under the influence of urbanization. Three cohorts studied differences between rural and urban populations, including several Tanzanian studies with 323 urban and hunter-gatherer participants, a South-African cohort with 170 rural and urban women, and a large Chinese cohort with 2678 participants in 63 rural and urban locations.^7–10^ The disappearing microbes in these studies have been previously referred to as Volatile and/or Associated Negatively with Industrialized Societies of Humans (VANISH) and included a range of microbes from the *Prevotellaceae*, *Succinivibrionaceae*, and *Spirochetes* families. Emerging microbes were referred to as Bloom or Selected in Societies of Urbanization/Modernization (BLoSSUM) and comprised *Bacteroidacae, Bifidobacteriacae*, and *Enterobacteriacae* families.^23,24^ With our machine learning analyses, we could confirm that most of these families are indeed present among the top predictors along the urbanization and migration axis. However, we observed many other taxonomic families among the differentially abundant microbes in our cohort, illustrating that the microbial shift is not limited to a few bacterial families. For instance, many ASVs from the *Lachnospiraceae* families could be found among top predictors in both our abundance and prevalence analyses and tended to be more prevalent and abundant in the rural community. A few studies that included agricultural populations, and as such were more comparable to our cohort than studies with hunter-gatherer populations, found a similarly diverse nature of the shift in gut microbiota composition using analyses at genus or family level.^6,10^ Summarizing, our analyses show that the gut microbiota composition undergoes a profound shift with urbanization and migration, broader than VANISH and BLoSSUM microbial groups, that is likely dependent of the degree of urbanization.

In contrast to previous studies, we did not find any *Spirochetes* spp. among the predictors. When focusing on the *Spirochetes* family, we found that, despite a rural-to-urban decrease in two *Spirochetes* ASVs, the abundance and prevalence of these ASVs was already very low in the rural Ghanaians, explaining relatively little of the observed gradient in composition. Of note, since urbanization is a continuous scale and context-specific, this hampers the direct comparison of results across other existing rural-urban studies, considering that rural and urban environments within a country can already be quite diverse. For instance, the rural communities in the Ashanti region are agricultural, which is more urbanized compared to hunter-gatherer populations in previous studies.^23,24,26^ Additionally, a microbial shift upon urbanization cannot be interpreted separately from the economic and sociocultural context. Ghana is a lower middle-income country (LMIC),^27^ which is economically vastly different from low income or, on the other end, upper middle-income countries in sub-Saharan Africa. Thus, some of the differences between our analyses and previous papers can be explained by the differences in sociocultural and economical context that are relevant in the context of gut microbiota composition.

We found the most pronounced differences in gut microbiota between urban Ghanaians and Amsterdam Ghanaians, corroborating the impact of migration on the gut microbiome, which was illustrated by the loss of *Weisella* spp. and a decrease in *Enterobacteriaceae* spp. There is a limited number of international migration studies on the gut microbiome, and to date, none describes migration from African to European countries.^11,14^ A previous study following six individuals that migrated from Thailand to the United States with sampling before and after the event, found that the gut microbiota changed substantially soon (within six to nine months) after migration, in parallel with an increase in BMI.^11^ In line with urbanization studies and our results, they found an increase in *Bacteroides* and concomitant decrease in *Prevotella* spp. This change was also observed in studies comparing migration generations.^14^ In our population, the role of *Prevotella* and *Bacteroides* was limited, and ASVs from families such as *Lactobacillaceae* (specifically, *Weisella* spp.), *Bifidobacteriaceae* and *Enterobacteriaceae* were more important in the machine learning models. While from rural to urban context differences in sanitation and exposure to animals might play a role, we speculate that the difference in food microbial exposures between Ghana and Amsterdam might be more relevant in a migration context, since *Lactobacillaceae* are also implicated in food fermentation.^28^

We also explored the associations between dietary intake and other established confounders of gut microbiota composition. Despite the relatively large study population, we found weak or absent associations of a range of covariates including age and sex.^10,29,30^ In contrast, the effect of BMI and several dietary macronutrients, especially protein and salt, on microbiota composition was substantial, aligning with previous reports.^31^ While the influence of protein intake on the gut microbiome is well-documented, the mechanisms underlying the impact of sodium intake remain relatively unexplored. In our regression models, we incorporated variables such as age, BMI and dietary intake, to evaluate the influence of these factors on the observed microbial differences. The rural-urban differences could partly be attributed to these covariates, emphasizing the role of host and dietary factors on the compositional shift.

Our study has several limitations, including its cross-sectional design. Longitudinal sampling of migrating individuals is useful to assess the effect of urbanization and migration, however, it is likely only feasible in small study populations due to the labor intense and costly organization of longitudinal cohorts. Although we adjusted our analyses for several relevant confounders to assess to what extent these explain the observed differences, there is likely residual confounding by unmeasured factors that are different across settings. These include living situations, sanitation, animal exposure, and environmental pollution, which could potentially affect health status, diet and gut microbiota composition. The differences between research sites are a reflection of a myriad of confounding factors, measured and unmeasured, that cannot easily be corrected by taking into account some of the covariates. Additionally, we focused on cardiometabolic risk factors and disease in the current study, while the microbiome could also affect other conditions of which data were not available, particularly those involving the immune system or gastrointestinal health (e.g. inflammatory bowel disease), which could be an interesting direction for future studies.

This population-based cohort study is unique as it is first large population-based cohort in West-Africa to study gut microbiota composition, including Ghanaians in different contexts in Ghana and in Europe, but originating from the same region. Every participant underwent detailed phenotyping, providing comprehensive information on health status and, importantly, dietary habits. To better understand the effects of the gut microbiota on blood pressure, the immune system and insulin resistance, studies focusing on urbanization and migration using longitudinal sampling are needed. In addition, shotgun metagenomics could improve our understanding of functional pathways and ecological dynamics in this population.

In conclusion, we observed a clear gradient in gut microbiota composition and a decrease in richness from rural Ghana, to urban Ghana and Amsterdam. Our abundance and prevalence analyses underlined that this shift affects microbes from a range of taxonomic families and is only partly explained by covariates such as diet. Unmeasured variables that include environmental exposures (food microbiome, animal exposure, sanitation) and sociocultural and economical context might play a role in the observed differences along the migration axis. The disappearance and emergence of microbes were associated with more disadvantageous cardiometabolic risk profiles, which, in the context of the existing literature, is likely a bidirectional association. Future research could therefore focus on improving gut health to influence cardiovascular outcomes.

## Supplementary Material

Supplemental Material
