# Supplementary material for: Gut microbiota shift in Ghanaian individuals along the migration axis: the RODAM-Pros cohort
Source: Gut Microbes. 2025 Apr 6;17(1):2471960. doi: 10.1080/19490976.2025.2471960 (PMC11980510; doi:10.1080/19490976.2025.2471960)
Supplement: Supplemental Material [file KGMI_A_2471960_SM5374.pdf]

SUPPLEMENTARY MATERIALS

Gut microbiota shift in Ghanaian individuals along the migration axis: the RODAM-Pros cohort

B.J.H. Verhaar,[1,2,3*] E.L van der Linden,[1,4] C.F. Hayfron-Benjamin,[1,5] E. Owusu-Dabo,[6] S.N. Darko,[7] S. Twumasi-Ankrah,[8] P. Henneman,[9] E. Beune,[1] K.A.C. Meeks,[1,10] M. Nieuwdorp,[2] H. Herrema,[3,11] B.J.H. van den Born,[1,2,3] C. Agyemang[1,4]

1 Department of Public and Occupational Health, Amsterdam UMC, location AMC, Amsterdam, The Netherlands.

2 Department of Vascular Medicine, Amsterdam UMC, location AMC, Amsterdam, The Netherlands.

3 Amsterdam Cardiovascular Sciences, Amsterdam UMC, Amsterdam, The Netherlands

4 Amsterdam Public Health, Amsterdam UMC, Amsterdam, The Netherlands

5 Department of Physiology, University of Ghana Medical School, Accra, Ghana

6 School of Public Health, Kwame Nkrumah University of Science and Technology (KNUST), Kumasi, Ghana.

7 Department of Molecular Medicine, Kwame Nkrumah University of Science and Technology (KNUST), Kumasi, Ghana.

8 Department of Statistics and Actuarial Science, Kwame Nkrumah University of Science and Technology (KNUST), Kumasi, Ghana.

9 Department of Human Genetics, Reproduction & Development, Amsterdam UMC, University of Amsterdam, Amsterdam, The Netherlands.

10 Center for Research on Genomics and Global Health, National Human Genome Research Institute, National Institutes of Health, Bethesda, Maryland, USA.

11 Department of Experimental Vascular Medicine, Amsterdam UMC, location AMC, Amsterdam, The Netherlands

* Corresponding author: Barbara J.H. Verhaar, MD PhD, Department of Vascular Medicine, Amsterdam UMC – Location AMC, PO box 22660, 1100 DD, Amsterdam, The Netherlands, E-mail: b.j.verhaar@amsterdamumc.nl

Supplementary Table 1: Machine learning models

A. Gut microbiota composition and health and dietary factors in the RODAM cohort

Name of model	Model type	Predictors	Outcome	Population
Gut microbiota and protein intake	Regression (continuous outcome)	Microbiota: all ASVs more prevalent than 10 counts in 30% of subjects	Protein intake	All participants
Gut microbiota and sodium intake	Regression (continuous outcome)	Microbiota: all ASVs more prevalent than 10 counts in 30% of subjects	Sodium intake	All participants
Gut microbiota and fat intake	Regression (continuous outcome)	Microbiota: all ASVs more prevalent than 10 counts in 30% of subjects	Fat intake	All participants
Gut microbiota and carbohydrate intake	Regression (continuous outcome)	Microbiota: all ASVs more prevalent than 10 counts in 30% of subjects	Carbohydrate intake	All participants
Gut microbiota and total calories	Regression (continuous outcome)	Microbiota: all ASVs more prevalent than 10 counts in 30% of subjects	Total calories	All participants
Gut microbiota and fibre intake	Regression (continuous outcome)	Microbiota: all ASVs more prevalent than 10 counts in 30% of subjects	Fibre intake	All participants
Gut microbiota and body mass index (BMI)	Regression (continuous outcome)	Microbiota: all ASVs more prevalent than 10 counts in 30% of subjects	BMI	All participants
Gut microbiota and age	Regression (continuous outcome)	Microbiota: all ASVs more prevalent than 10 counts in 30% of subjects	Age	All participants
Gut microbiota and low-density lipoprotein (LDL) levels	Regression (continuous outcome)	Microbiota: all ASVs more prevalent than 10 counts in 30% of subjects	LDL levels	All participants
Gut microbiota and C-reactive protein (CRP)	Regression (continuous outcome)	Microbiota: all ASVs more prevalent than 10 counts in 30% of subjects	CRP levels	All participants
Gut microbiota and estimated glomerular	Regression (continuous outcome)	Microbiota: all ASVs more prevalent than 10	eGFR	All participants

filtration rate (eGFR)		counts in 30% of subjects		
Gut microbiota and hypertension	Classification (binary outcome)	Microbiota: all ASVs more prevalent than 10 counts in 30% of subjects	Hypertension (yes/no)	All participants
Gut microbiota and sex	Classification (binary outcome)	Microbiota: all ASVs more prevalent than 10 counts in 30% of subjects	Sex (male/female)	All participants
Gut microbiota and antihypertensive drugs	Classification (binary outcome)	Microbiota: all ASVs more prevalent than 10 counts in 30% of subjects	Antihypertensive drugs (yes/no)	All participants

B. Predicting geographical site from gut microbiota composition (abundance)

**Name of model**	**Model type**	**Predictors**	**Outcome**	**Population**
Gut microbiota in rural versus urban Ghana	Classification (binary outcome)	Microbiota: all ASVs more prevalent than 10 counts in 30% of subjects	Location (rural / urban Ghana)	Rural Ghana and urban Ghana participants
Gut microbiota in urban Ghana versus Amsterdam	Classification (binary outcome)	Microbiota: all ASVs more prevalent than 10 counts in 30% of subjects	Location (urban / Amsterdam)	Urban Ghana and Amsterdam participants

C. Predicting geographical site from gut microbiota composition (presence)

**Name of model**	**Model type**	**Predictors**	**Outcome**	**Population**
Gut microbiota in rural versus urban Ghana	Classification (binary outcome)	Microbiota presence-absence matrix of ASVs prevalent in 10% of subjects	Location (rural / urban Ghana)	Rural Ghana and urban Ghana participants
Gut microbiota in urban Ghana versus Amsterdam	Classification (binary outcome)	Microbiota presence-absence matrix of ASVs prevalent in 10% of subjects	Location (urban / Amsterdam)	Urban Ghana and Amsterdam participants

Supplementary Table 2: Linear regression models

Name of model	Predictors	Outcome	Covariates	Population
Rural-vs-urban	Geographical location (rural vs urban)	Microbiota: top 20 features from XGBoost model rural vs urban Ghana	Model 0: unadjusted<br>Model 1: age, sex, BMI, hypertension<br>Model 2: model 1 covariates and PC1 and PC2 of dietary macronutrient groups	Rural and urban Ghana participants
Urban-vs-Amsterdam	Geographical location (urban vs Amsterdam)	Microbiota: top 20 features from XGBoost model urban Ghana vs Amsterdam	Model 0: unadjusted<br>Model 1: age, sex, BMI, hypertension<br>Model 2: model 1 covariates and PC1 and PC2 of dietary macronutrient groups	Urban Ghana and Amsterdam participants

Supplementary Figure 1: XGBoost machine learning model design

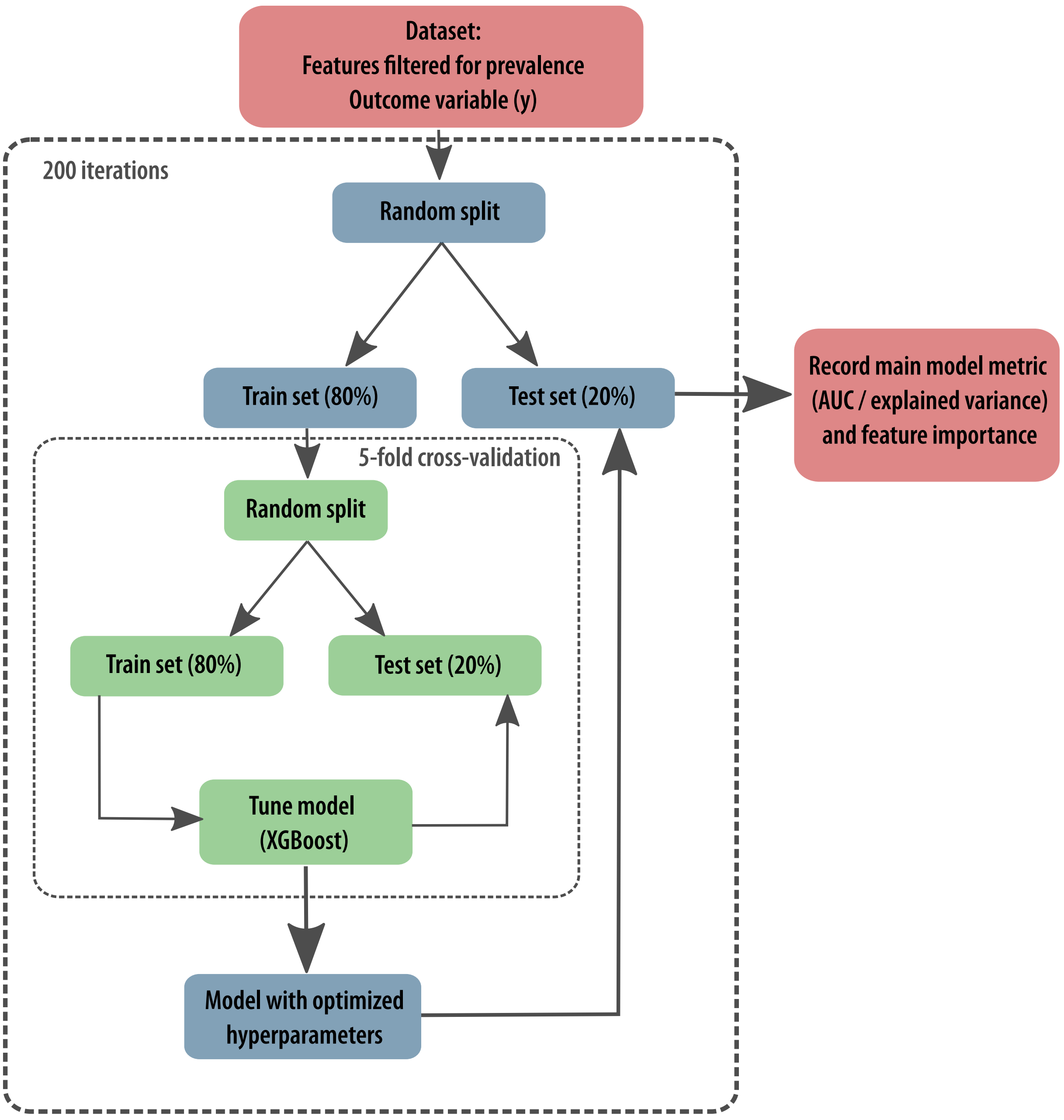

Supplementary Figure 2: Dietary intake

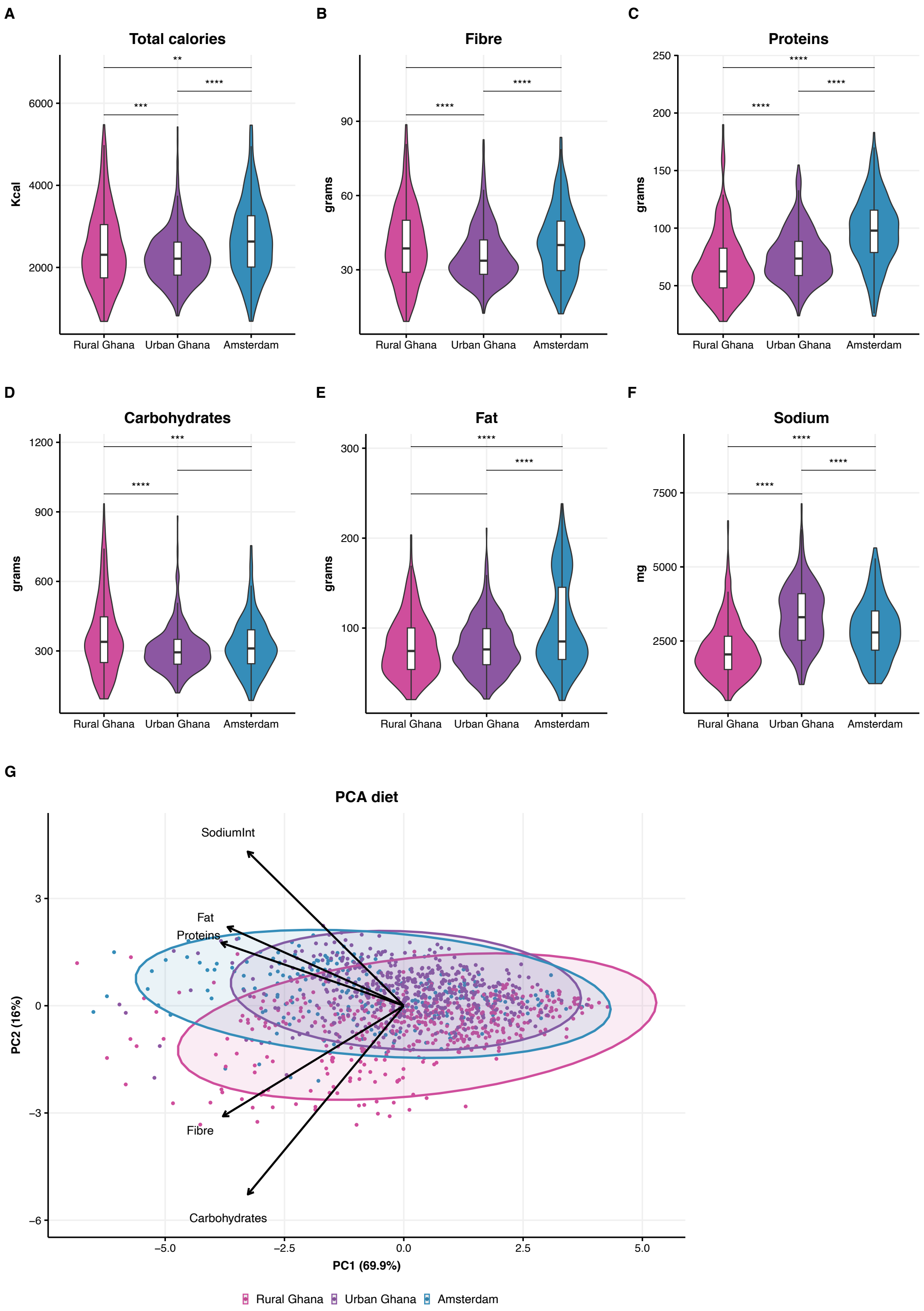

Differences in dietary intake between rural Ghana, urban Ghana, and Amsterdam. The violin plots show differences in total calorie intake (A), fibre intake (B), protein intake (C), carbohydrate intake (D), fat intake (E), and sodium intake (F). The principal component analysis (PCA) plot (G) illustrates the variation in these macronutrient groups, summarized into two dimensions. The first two principal components (PCs) were obtained using the prcomp function in R, with dietary variables scaled to zero mean and unit variance. The arrows indicate the loadings of the dietary variables in the two-dimensional space, showing their contribution to the principal components.

Supplementary Figure 3: Dietary intake: vanish group versus controls

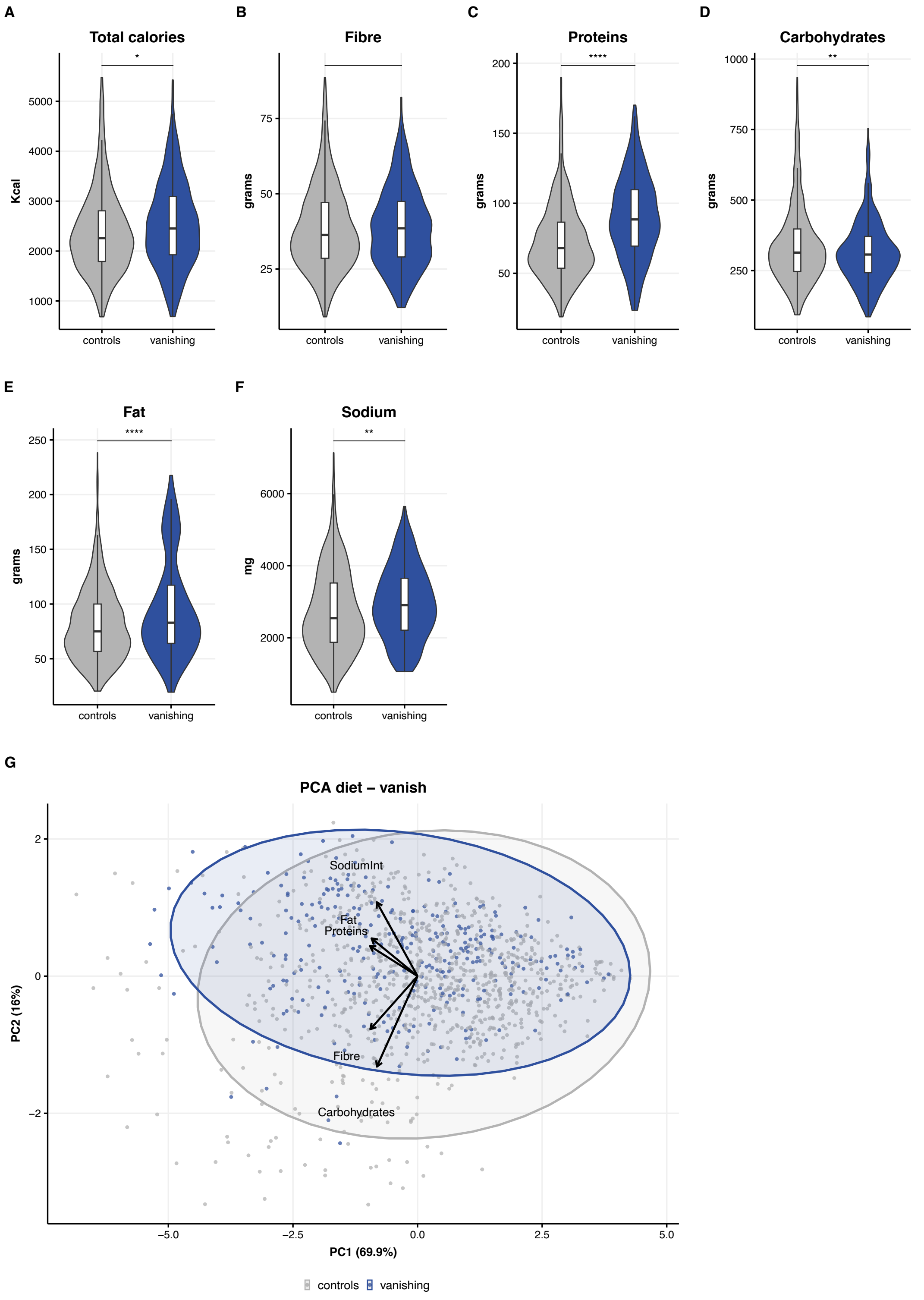

Differences in dietary intake between the vanishing group and controls. The violin plots show differences in total calorie intake (A), fibre intake (B), protein intake (C), carbohydrate intake (D), fat intake (E), and sodium intake (F). The principal component analysis (PCA) plot (G) illustrates the variation in these macronutrient groups, summarized into two dimensions. The first two principal components (PCs) were obtained using the prcomp function in R, with dietary variables scaled to zero mean and unit variance. The arrows indicate the loadings of the dietary variables in the two-dimensional space, showing their contribution to the principal components.

Supplementary Figure 4: Dietary intake: blossom group versus controls

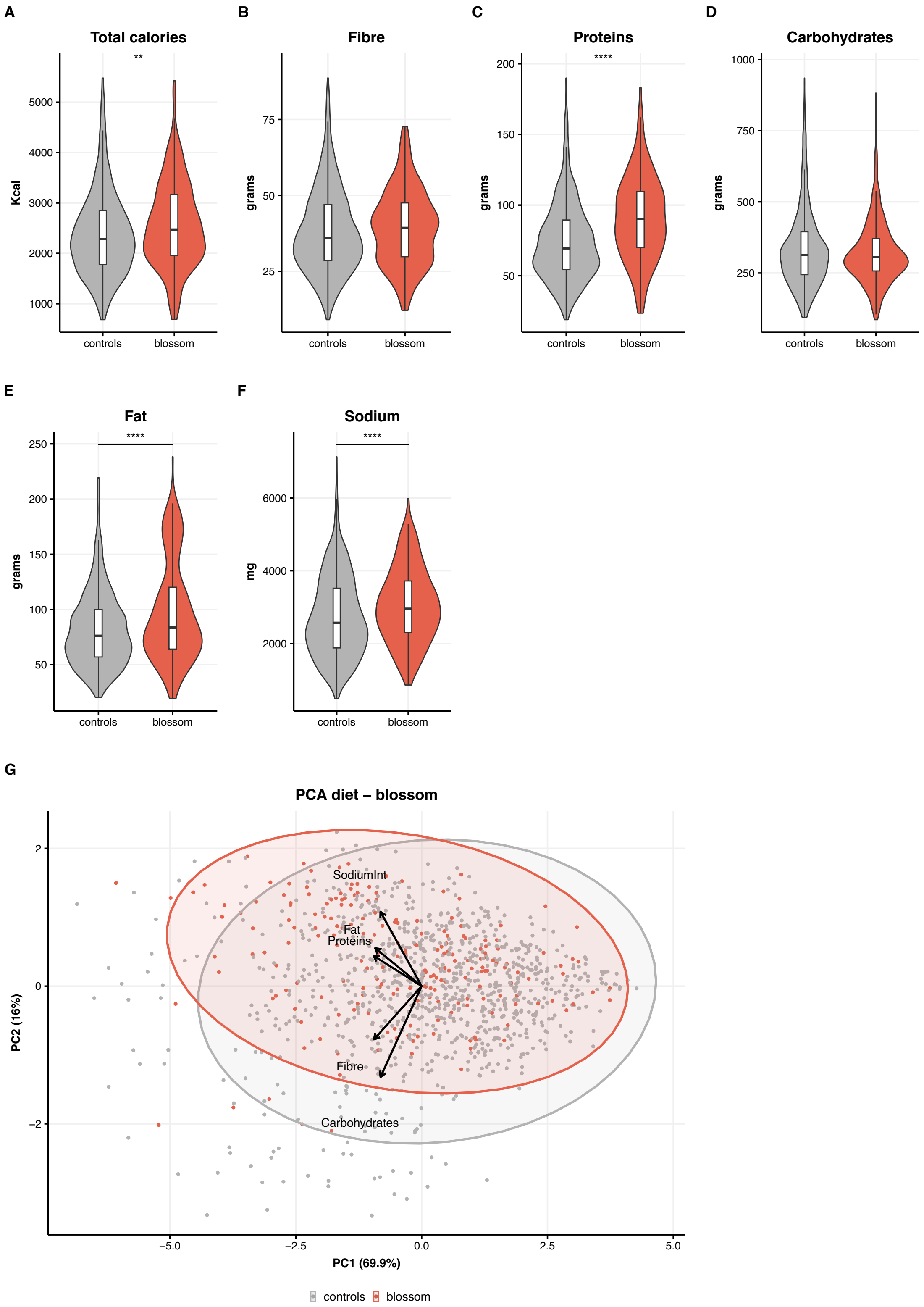

Differences in dietary intake between the vanishing group and controls. The violin plots show differences in total calorie intake (A), fibre intake (B), protein intake (C), carbohydrate intake (D), fat intake (E), and sodium intake (F). The principal component analysis (PCA) plot (G) illustrates the variation in these macronutrient groups, summarized into two dimensions. The first two principal components (PCs) were obtained using the prcomp function in R, with dietary variables scaled to zero mean and unit variance. The arrows indicate the loadings of the dietary variables in the two-dimensional space, showing their contribution to the principal components.
